# Transcolonic Migration of Retained Epicardial Pacing Wires

**DOI:** 10.1155/2015/416587

**Published:** 2015-06-18

**Authors:** Sara Gonzales, Hugh White, Juan Echavarria

**Affiliations:** ^1^Department of Radiology, University of Texas Health Science Center at San Antonio, 7703 Floyd Curl Drive, San Antonio, TX 78229, USA; ^2^Department of Gastroenterology, University of Texas Health Science Center at San Antonio, 7703 Floyd Curl Drive, San Antonio, TX 78229, USA

## Abstract

Temporary epicardial pacing wires are associated with rare complications. Most of these occur in the chest. Even rarer are complications that occur within the abdomen. We report a case of migrating epicardial pacing wires entering the abdomen and penetrating the transverse colon found incidentally on colonoscopy in an asymptomatic patient.

## 1. Introduction

Temporary epicardial pacing wires may be used after cardiac surgery in the treatment of arrhythmias. Although these wires are typically removed prior to hospital discharge, if there is resistance during attempts at removal, they are typically cut flush with the skin and allowed to retract into the pericardial sac. These residual epicardial wires may result in rare complications, most commonly within the chest. We report a case of migrating epicardial pacing wires entering the abdomen and penetrating the transverse colon found incidentally on colonoscopy in an asymptomatic patient.

## 2. Clinical Presentation

**Figure 1:**
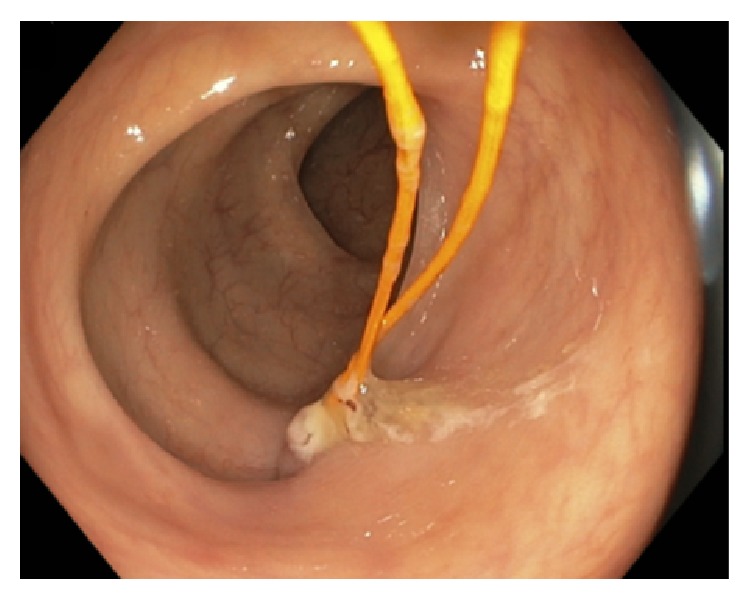


**Figure 2 fig2:**
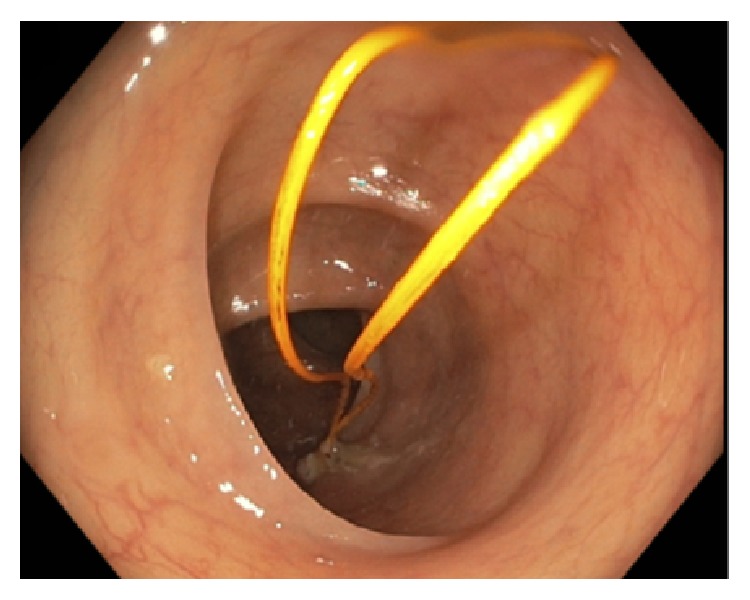


**Figure 3:**
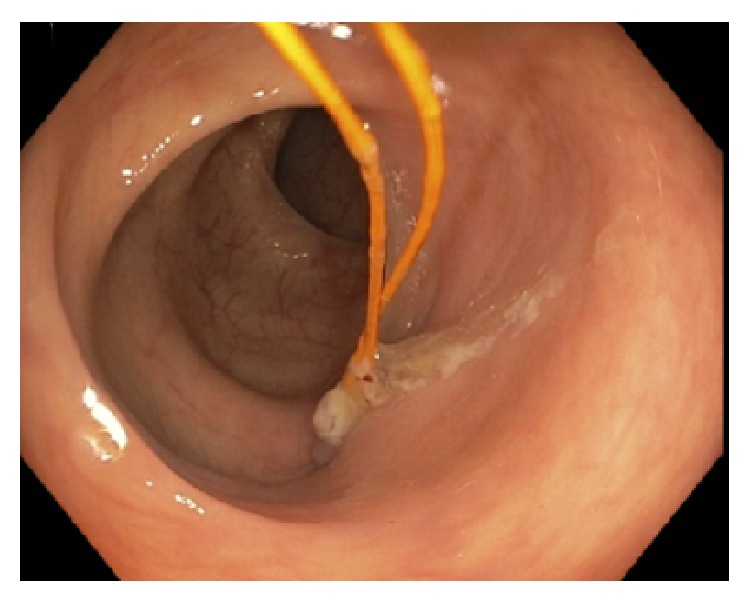


**Figure 4:**
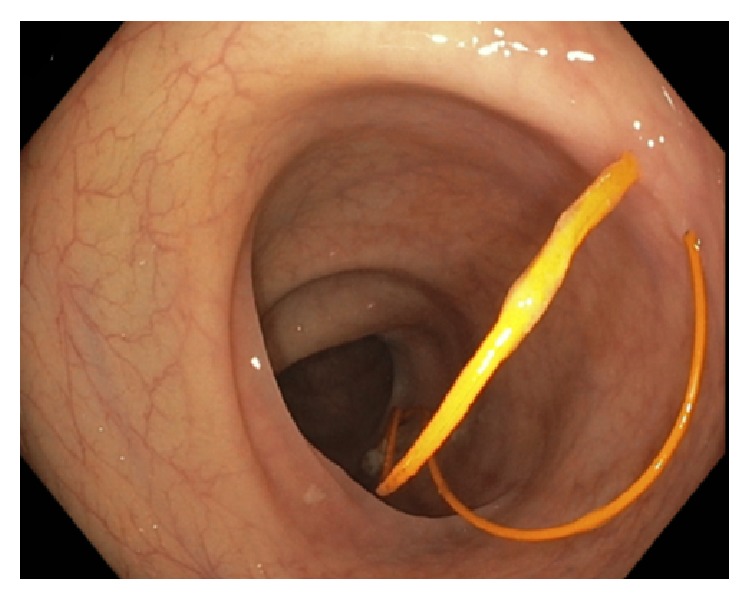


**Figure 5:**
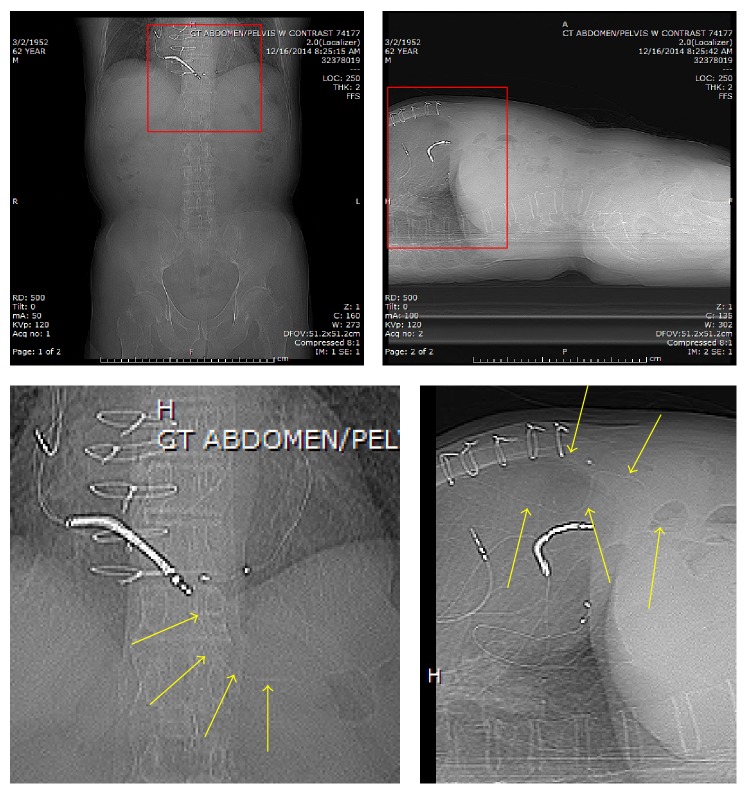
Scout images demonstrating faint linear hyperdensities (yellow arrows) vertically oriented descending below the diaphragm. The lateral scout view demonstrates the distal most aspect of the wires overlying air-filled transverse colon (found to be intraluminal on the following CT).

**Figure 6 fig6:**
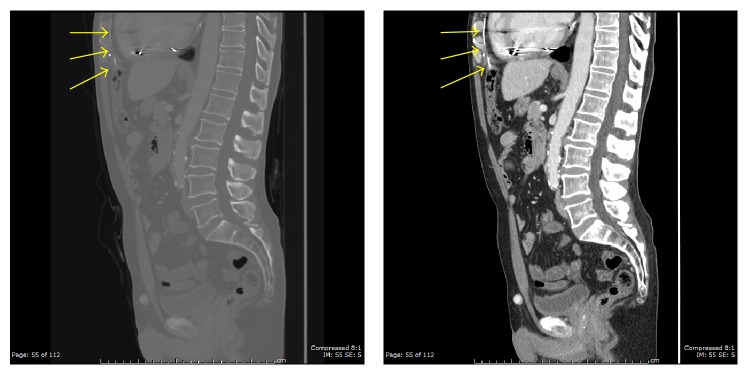
Sagittal images in bone and soft tissue windows revealing hyperdense wire (yellow arrows) vertically oriented in the substernal space traversing the sternocostal triangle (foramen of Morgagni) and piercing the transverse colon wall.

**Figure 7:**
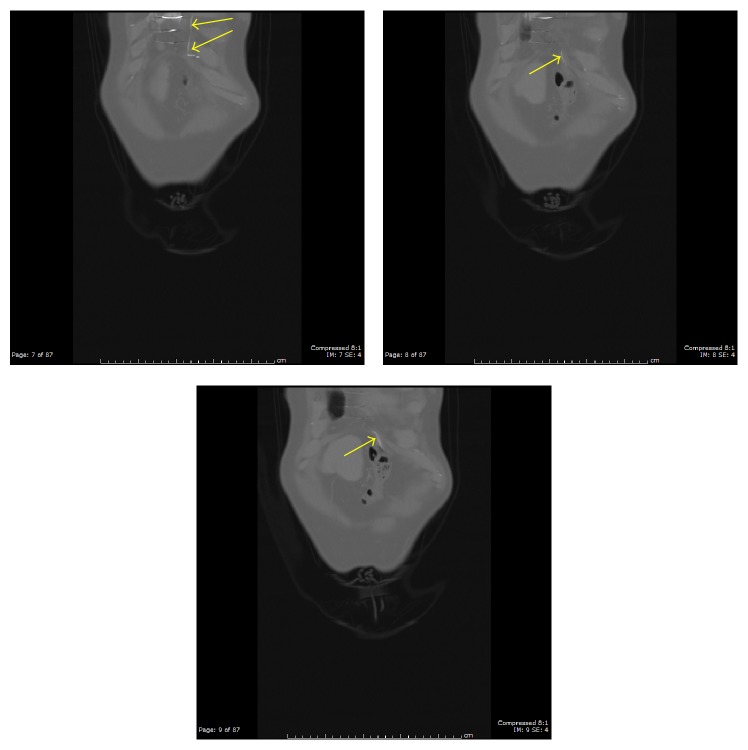
Coronal images with bone window following the course of the migrating epicardial pacing wire (yellow arrows).

**Figure 8:**
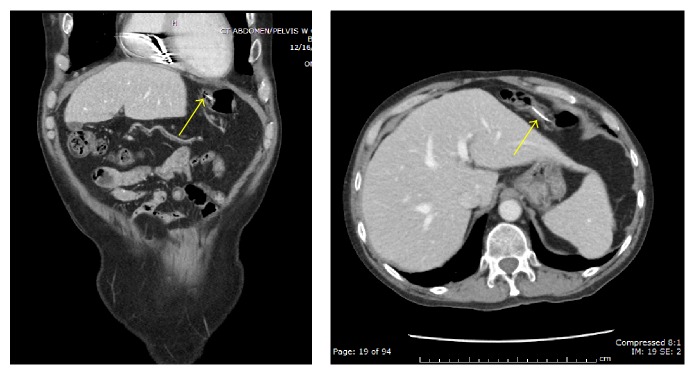
Coronal and axial images in soft tissue window revealing endoluminal extension of the migrating epicardial pacing wiring into the transverse colon (yellow arrows).

A 62-year-old male with a past medical history of heart disease status after coronary artery bypass grafting (CABG) in August 2010 and implantable cardioverter defibrillator placement in April 2014 presented for a routine screening colonoscopy in December 2014. During screening colonoscopy, two yellow wires were seen within the lumen of the colon (Figures 1 [Fig fig2]–3). Per colonoscopy report, “one end of both wires was moving loosely in the lumen, whereas the other end of both wires seems to be protruding externally into the colon through the mucosa” (Figure 4). Once the wires were identified within the colon, the colonoscopy was aborted. The patient was asymptomatic at the time, and CT abdomen/pelvis was ordered for further evaluation. CT demonstrated two migrating epicardial pacing wires (one in the substernal space and one along the right heart border) joining together before passing through the sternocostal triangle, entering the abdomen, and penetrating the transverse colon wall (Figures 5 [Fig fig6]–7). An additional linear metallic fragment within the transverse colon was thought to represent a disconnected migrating segment of an epicardial pacing wire (Figure 8). No associated focal fluid collection, free air, or inflammatory changes were identified. The physician who performed the CABG four years earlier was contacted, who reported that after unsuccessful attempts at removing the wires, the wires were cut at the skin surface. As this is a rare situation without clear guidelines for treatment, the patient was referred for a cardiothoracic surgery consultation. As the patient was asymptomatic, surgical intervention was not pursued.

## 3. Discussion

Temporary epicardial pacing wires have been routinely used in the management of patients who have undergone recent cardiac surgery. The main indication for their insertion is perioperative arrhythmias, which may result in significant hemodynamic compromise. These wires may be helpful in the operating room as well as in the immediate postoperative period to optimize cardiac function. Epicardial pacing wires are typically placed at the end of the cardiac procedure, at which time atrial pacing wires are sutured to the right atrial appendage or body of the right atrium and ventricular pacing wires are placed on the anterior or diaphragmatic surface of the right ventricle. Left ventricular electrodes may be placed in the apex and then passed percutaneously to the left of midline and secured [1]. Epicardial pacing wires are generally removed on the fourth postoperative day. Wires are removed with gentle traction. If resistance is met while attempting to remove the epicardial wires, the wires may be cut flush with the skin so that the residual wire retracts into the tissue [2].

Serious but infrequent complications can be seen with temporary epicardial pacing wires. Complications can either occur at the time of surgery or shortly thereafter, including during removal. Bleeding can occur from the site of insertion. During wire removal, complications include hemorrhage and tamponade from atrial and ventricular lacerations, injuries to saphenous vein grafts, and retained fragments. Permanent wire fragments secondary to inability to remove wires are most often asymptomatic, but late complications have also been reported including fracture, migration, or infection. Migration of the wires into the right ventricle, right ventricular outflow tract, left pulmonary artery, bronchial system, ascending aorta, and right carotid artery has all been reported. Review of the literature reveals only three previously reported cases of intraoperative colon perforation by temporary pacing wires, all discovered in the first week after surgery [3–5]. To our knowledge the case presented here is the first reported case of late discovery of colonic perforation by epicardial pacing wires. To be fair, it is unclear if the case present here was similar to reported cases in which perforation occurred at the time of surgery or if there was chronic migration and penetration of the colonic wall by the temporary pacing wires. The patient's reportedly uneventful postoperative course suggests that intraoperative perforation was unlikely and an insidious process of chronic migration is more likely. Although rare, the radiologist should be aware of potential complications of temporary epicardial pacing wires, including intra-abdominal migration or misplacement, so as to exclude any of these complications when reviewing imaging examinations.
